# Association Between Serum Lipid-Bilirubin Ratio and Clinical Prognosis Among Patients Undergoing Percutaneous Coronary Intervention After Coronary Artery Bypass Grafting

**DOI:** 10.31083/RCM39827

**Published:** 2025-11-13

**Authors:** Xu Yan, Muhib ur Reheman, Qiuxuan Li, Zhiqiang Yang, Lixia Yang, Zhijian Wang, Yujie Zhou

**Affiliations:** ^1^Department of Cardiology, Beijing Anzhen Hospital Affiliated to Capital Medical University, 100029 Beijing, China

**Keywords:** percutaneous coronary intervention, MACCE, LDL-C/(HDL-C+DBIL), *in situ* blood vessels, bridging vessel

## Abstract

**Background:**

The low-density lipoprotein cholesterol (LDL-C)/(high-density lipoprotein C (HDL-C) + direct bilirubin (DBIL)) ratio has been linked to the development of atherosclerosis. However, the association of this ratio with clinical outcomes in patients with prior coronary artery bypass grafting (CABG) undergoing percutaneous coronary intervention (PCI) remains unclear. Therefore, this study aimed to explore whether the LDL/(HDL + DBIL) ratio is predictive of clinical outcomes in this patient group.

**Methods:**

We retrospectively reviewed 1352 patients who underwent re-PCI after CABG surgery and categorized the patients into three groups based on the third quartile of the ratio levels. The primary endpoint was major adverse cardiovascular and cerebrovascular events (MACCE), defined as a composite of all-cause death, stroke, myocardial infarction, or target vessel revascularization.

**Results:**

During the follow-up period, the occurrence rate of MACCE in the high ratio group was significantly higher than that in the low to moderate ratio groups (9.9% vs. 11.4% vs. 20.1%; *p* < 0.001). This trend was consistent for cardiac death (6.2% vs. 6.2% vs. 9.8%; *p* = 0.021) and non-fatal myocardial infarction (3.2% vs. 4.0% vs. 7.4%; *p* = 0.003). After adjusting for other risk factors, Cox multiple regression analysis suggested that LDL-C/(HDL-C + DBIL) remained significantly correlated with MACCE (hazard ratio (HR) = 1.33, 95% confidence interval (CI): 1.186–1.193; *p* < 0.001) with the high ratio group having the highest risk (HR = 2.331, 95% CI: 1.585–3.427; *p* < 0.001). According to the subgroup analysis, the selection of bypass graft or native vascular PCI did not affect the relationship between the ratio and the occurrence of MACCE.

**Conclusion:**

The LDL-C/(HDL-C + DBIL) ratio level is closely related to the risk of long-term MACCE in patients undergoing PCI after CABG surgery, and the LDL-C/(HDL-C + DBIL) level can be an important indicator for post-PCI risk assessment.

## 1. Introduction

Current coronary artery revascularization techniques primarily involve coronary artery bypass grafting (CABG) and percutaneous coronary intervention (PCI) [1]. CABG serves as the primary treatment for patients with triple-vessel coronary artery disease and/or left main coronary artery disease, particularly in diabetic patients. However, graft vessel (GV) failure, particularly in saphenous vein grafts (SVGs), is common post-CABG, with failure rates of SVGs reaching 15%–20% at 1 year and approximately 50% at 10 years post-surgery [2]. Patients with a history of CABG often experience rapid progression of atherosclerotic lesions in native coronary artery vessels (NVs) and GVs, leading to recurrent angina or acute coronary syndrome (ACS) events [3, 4]. Despite optimal medical therapy, satisfactory clinical outcomes are often elusive, necessitating repeat coronary artery revascularization to ameliorate symptoms [5]. However, compared to primary CABG, patients undergoing repeat CABG face higher mortality rates and poorer prognoses, particularly due to advanced age and comorbidities [6]. Consequently, PCI emerges as the preferred revascularization strategy for patients with a history of CABG [7].

Recent research has increasingly focused on novel biomarkers such as low-density lipoprotein cholesterol (LDL-C), high-density lipoprotein cholesterol (HDL-C), and direct bilirubin (DBIL) [8, 9, 10]. LDL-C contributes to the development of atherosclerotic plaques, vascular inflammation, and endothelial cell damage, thereby elevating the risk of major adverse cardiovascular and cerebrovascular events (MACCE) in patients with coronary artery disease (CAD) [11]. Conversely, HDL-C exerts diverse beneficial effects by facilitating cholesterol efflux and diminishing cholesterol accumulation in arterial walls [12]. Moreover, HDL-C suppresses inflammation, enhances endothelial function, and displays antioxidant properties, collectively safeguarding endothelial cell integrity and decreasing the risk of plaque rupture [13, 14, 15]. DBIL inhibits oxidative stress and inflammation, reducing free radical production and protecting endothelial cell integrity, thereby lowering plaque rupture and thrombosis risk [16]. Furthermore, DBIL possesses antiplatelet and anticoagulant properties, further mitigating MACCE risk in CAD patients [17].

To enhance assessment accuracy, researchers have proposed the concept of LDL-C/(HDL-C+DBIL) ratio. This ratio integrates the risk factor LDL-C with the protective factors HDL-C and DBIL, accounting for the integrated effects of various potential mechanisms, including cholesterol metabolism, oxidative stress, and inflammation [18]. A retrospective analysis of data from Chinese CAD patients published in 2020 found a significant correlation between LDL-C/(HDL-C+DBIL) ratio and MACCE incidence, with higher ratios associated with increased MACCE risk and lower ratios correlated with reduced risk [19]. This study provided initial evidence for further exploring the relationship between LDL-C/(HDL-C+DBIL) ratio and MACCE and analyzing its role in pre-PCI risk assessment. Additionally, it offers valuable insights into repeat PCI treatment for patients with a history of CABG and explores the potential clinical application of LDL-C/(HDL-C+DBIL) ratio.

## 2. Materials and Methods

### 2.1 Study Population

**Fig. 1.**
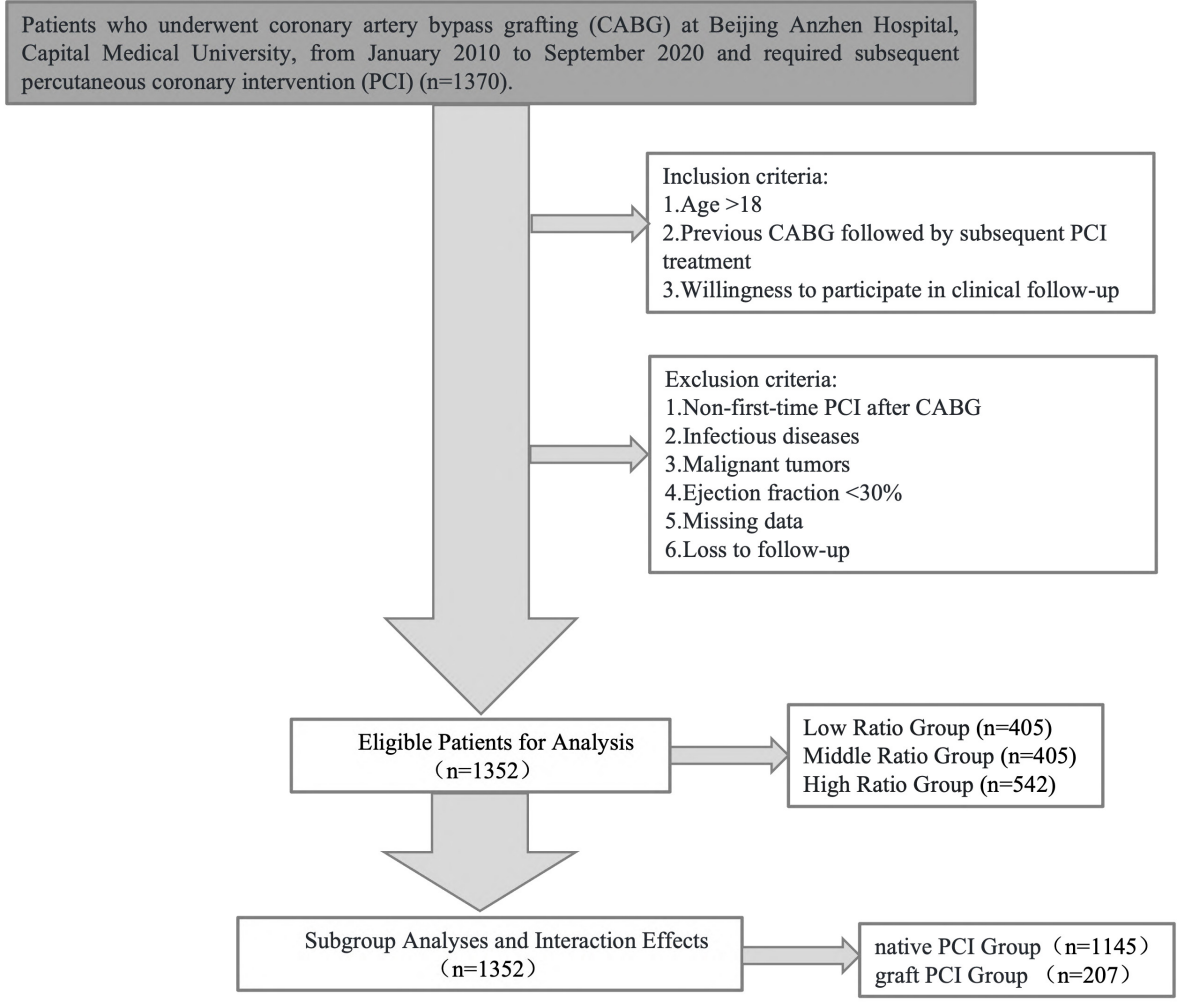
**Flowchart of this study**.

This study is an observational, retrospective study based on the National Clinical Research Center for Cardiovascular Diseases (Beijing Anzhen Hospital, Beijing, China). The study analyzed a total of 1352 eligible patients who underwent PCI for the first time after CABG at Beijing Anzhen Hospital affiliated to Capital Medical University from January 2010 to September 2020. This study was approved by the Ethics Committee of Beijing Anzhen Hospital affiliated to Capital Medical University and was conducted in accordance with the principles outlined in the Helsinki Declaration (Fig. 1).

### 2.2 Inclusion Criteria

(1) Age >18 years;

(2) Previous CABG followed by subsequent PCI treatment;

(3) Willingness to participate in clinical follow-up.

### 2.3 Exclusion Criteria

(1) Non-first-time PCI after CABG;

(2) Infectious diseases;

(3) Malignant tumors;

(4) Ejection fraction <30%;

(5) Missing data;

(6) Loss to follow-up.

### 2.4 Grouping of Patients

(1) Patients were stratified based on their lipid profiles and bilirubin levels:

∙ Low ratio group: LDL-C/HDL-C + DBIL <1.82 (n = 405);

∙ Middle ratio group: 1.82 ≤ LDL-C/HDL-C + DBIL < 2.44 (n = 405);

∙ High ratio group: LDL-C/HDL-C + DBIL ≥2.44 (n = 542).

(2) Patients were also classified based on the type of PCI performed:

∙ Native PCI group (n = 1145): This group included patients who underwent PCI on their native coronary arteries.

∙ Graft PCI group (n = 207): This group consisted of patients who underwent PCI on graft vessels.

Notably, patients who received both native vessel PCI and graft vessel PCI were categorized into the graft PCI group for analysis purposes. No patients underwent both left internal mammary artery graft PCI and saphenous vein graft PCI (SVG-PCI).

### 2.5 Definition of Covariates

Data was collected retrospectively from medical records, including demographic information, clinical characteristics, and procedural details. Body mass index (BMI) was calculated using the formula: weight (kg)/height^2^ (m^2^). Coronary angiography and PCI were conducted using radial and/or femoral artery access through the standard Judkins technique. Angiography was performed in at least two views to assess the left main coronary artery, left anterior descending artery, left circumflex artery, and right coronary artery. Coronary artery lesions were defined by a visual estimation of greater than 50% diameter stenosis. Selection of native vessel or graft vessel for PCI treatment was determined by the surgical team based on angiographic results and surgical risks. Follow-up data were obtained through outpatient visits or by contacting patients directly.

### 2.6 Laboratory Tests and Examinations

All patients had fasting venous blood drawn for laboratory tests after admission, including measurements of DBIL high-density lipoprotein (HDL), low-density lipoprotein (LDL), triglycerides (TG), total cholesterol (TC), fasting blood glucose, glycated hemoglobin, serum creatinine, and other laboratory indicators at the Central Laboratory of Beijing Anzhen Hospital affiliated to Capital Medical University. Left ventricular ejection fraction was measured by the echocardiography team at Beijing Anzhen Hospital. To calculate the bilirubin-lipid composite index LDL-C/(HDL-C+DBIL), the obtained DBIL units were converted to mmol/L and then combined with HDL-C, i.e., LDL-C (mmol/L)/[HDL-C (mmol/L)+DBIL (µmol/L)/1000].

### 2.7 Long-Term Follow-up

The primary endpoint was MACCE, defined as a composite of all-cause death, nonfatal stroke, nonfatal myocardial infarction, or target vessel revascularization (TVR). Secondary endpoints included cardiac death, all-cause death, nonfatal stroke, nonfatal myocardial infarction, and TVR. Myocardial infarction was defined as elevated levels of cardiac troponin or creatine kinase with ischemic symptoms or indicative electrocardiographic changes. The presence of new pathological Q waves in ≥2 contiguous leads was also diagnosed as myocardial infarction. Nonfatal stroke was defined as ischemic stroke with evidence of neurological dysfunction requiring hospitalization and documented lesions on brain computed tomography or magnetic resonance imaging. If stroke, myocardial infarction, or TVR occurred more than once, the most severe event (death > stroke > myocardial infarction > TVR) occurring for the first time was recorded as the clinical endpoint event, along with the time of occurrence. Endpoint events were adjudicated independently by at least two cardiologists.

### 2.8 Statistical Analysis

Data Analysis Statistical analysis was performed using SPSS (version 24.0; IBM Corporation, Armonk, NY, USA) and R Programming Language (version 4.1.0; R Foundation for Statistical Computing, Vienna, Austria). Normality of continuous variables was tested, with absolute values of skewness and kurtosis <3 considered to follow a normal distribution. Normally distributed continuous variables were presented as mean ± standard deviation. Between-group comparisons were made using the independent samples *t* -test for two groups and one-way ANOVA for three or more groups. Non-normally distributed continuous variables were presented as median (interquartile range), and between-group comparisons were made using Mann-Whitney U non-parametric test. Categorical variables were presented as frequencies and percentages, and between-group comparisons were made using chi-square test or Fisher’s exact probability test. All study patients were divided into native vessel group and graft vessel group based on the target vessel of PCI, and then the occurrence of long-term adverse cardiovascular events between groups was compared. Kaplan-Meier survival curves were used to compare differences in clinical events among different LDL-C/(HDL-C+DBIL) groups, and log-rank test was used for inter-group difference analysis. Cox multivariate regression model was applied to correct confounding factors associated with outcomes. The adjusted variables included demographic data (age, gender, body mass index, systolic blood pressure), cardiovascular risk factors (hypertension, dyslipidemia, diabetes, history of prior myocardial infarction, history of prior PCI, heart failure, history of stroke, chronic kidney disease, family history of coronary heart disease, clinical diagnosis [stable coronary artery disease (SCAD), unstable angina (UA), non-ST elevation myocardial infarction (NSTEMI) and ST elevation myocardial infarction (STEMI)], admission examination (HDL, LDL, triglycerides, total cholesterol, BMI, and LDL-C/(HDL-C+DBIL)), medication with drug-eluting stents (DES) implanted in each patient, time interval between CABG and PCI, and medication at discharge. LDL-C/(HDL-C+DBIL) was included in the model as both a continuous variable and a tertile categorical variable to analyze.

## 3. Results

### 3.1 Patient Characteristics

**Fig. 2.**
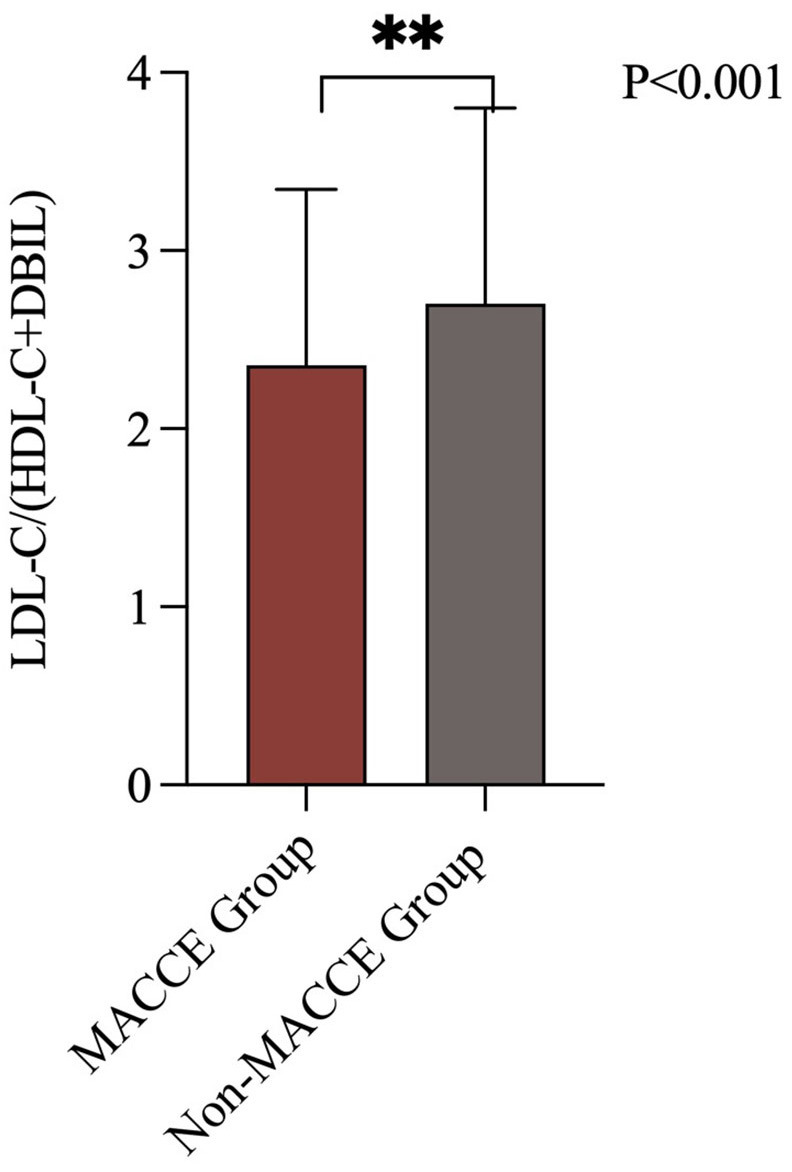
**LDL-C/(HDL-C+DBIL) levels in patients with and without MACCE**. MACCE, major adverse cardiac and cerebrovascular events; LDL-C, low-density lipoprotein cholesterol; HDL-C, high-density lipoprotein cholesterol; DBIL, direct bilirubin. The symbol ** indicates a *p*-value less than 0.001.

We analyzed a total of 1352 patients from January 2010 to September 2020, among whom 195 patients experienced MACCE events (Table 1). It was observed that the median age of the patients was 65 years (range: 59–70 years), with median ages of 64 years (range: 59–69 years) for the non-MACCE group and 67 years (range: 62–72 years) for the MACCE group. The age of patients in the MACCE group was significantly higher than that of patients in the non-MACCE group (*p* < 0.001). However, there were no significant differences in gender and weight between the two groups. Further analysis of risk factors revealed that the occurrence rates of hypertension, hyperlipidemia, and diabetes were not significantly different between the MACCE and non-MACCE groups. However, the incidence rates of myocardial infarction (MI) history (*p* = 0.024) and chronic kidney disease (CKD) (*p* = 0.001) were significantly higher in the MACCE group than in the non-MACCE group. Upon admission, the median levels of LDL and TC for the overall patients were 2.2 (1.8––2.8) and 3.8 (3.3––4.5), respectively, with median levels of 2.2 (1.7––2.7) for LDL and 3.8 (3.3––4.5) for TC in the non-MACCE group, and 2.4 (1.9––3.2) for LDL and 4.1 (3.5––4.8) for TC in the MACCE group. Significant differences in LDL and TC levels were observed between the two groups (*p* < 0.001). Of particular note, the median LDL-C/(HDL-C+DBIL) ratio for all patients was 2.2 (1.7––2.8), with median ratios of 2.2 (1.7––2.8) for the non-MACCE group and 2.6 (1.9––3.4) for the MACCE group, indicating a significantly higher level in the MACCE group compared to the non-MACCE group (*p* < 0.001) (Fig. 2). There were no similar significant differences in the examination results for HDL, TG, and BMI between the two groups. Based on clinical diagnosis data, it was observed that the odds of MACCE occurrence were similar among patients with different diagnosis types (SCAD, UA, NSTEMI, and STEMI), suggesting that clinical diagnosis type may not have a significant impact on MACCE occurrence. Regarding medication at discharge, no significant differences were found between the MACCE and non-MACCE groups in the use of statins, aspirin, P2Y12 receptor antagonists, or angiotensin II receptor blocker (ARB) or angiotensin receptor-neprilysin inhibitor (ARNI).

**Table 1.** **Baseline characteristics of the cohort**.

Category		All patients (n = 1352)	No MACCE (n = 1157)	MACCE (n = 195)	*p*-value
Demographics					
	Age (years)	65 (59–70)	64 (59–69)	67 (62–72)	<0.001
	Male sex, n (%)	988 (73.1)	834 (72.1)	154 (79.0)	0.725
	Weight (kg)	72 (65–80)	72 (65–80)	72 (64–80)	0.234
	Systolic BP (mmHg)	130 (119–140)	130 (119–140)	130 (120–140)	0.083
	BMI	26 (24–28)	26 (24–28)	26 (24–28)	0.925
Risk factors, n (%)					
	Hypertension	984 (72.8)	834 (72.1)	141 (72.3)	0.907
	Hyperlipidemia	1346 (99.6)	1153 (99.7)	194 (99.5)	0.567
	Diabetes	638 (47.2)	534 (46.2)	102 (52.6)	0.072
	History of MI	672 (49.8)	560 (48.4)	111 (57.2)	0.024
	Heart failure	93 (6.9)	827 (7.1)	12 (6.1)	0.603
	Stroke history	167 (12.4)	138 (11.9)	29 (15.0)	0.389
	Chronic kidney disease	53 (3.9)	36 (3.1)	16 (8.0)	0.001
	Family history of CAD	128 (9.5)	110 (9.5)	18 (9.4)	0.957
Admission examination					
	HDL mmol/L	1.0 (0.9–1.1)	1.0 (0.9–1.1)	1.0 (0.8–1.1)	0.139
	LDL mmol/L	2.2 (1.8–2.8)	2.2 (1.7–2.7)	2.4 (1.9–3.2)	<0.001
	TG mmol/L	1.5 (1.1–2.0)	1.5 (1.1–2.0)	1.5 (1.1–2.0)	0.658
	TC mmol/L	3.8 (3.3–4.5)	3.8 (3.3–4.5)	4.1 (3.5–4.8)	<0.001
	DBIL mmol/L	3.0 × 10^–⁢3^ (2.2 × 10^–⁢3^–4.1 × 10^–⁢3^)	3.0 × 10^–⁢3^ (2.2 × 10^–⁢3^–4.1 × 10^–⁢3^)	2.7 × 10^–⁢3^ (2.0 × 10^–⁢3^–4.1 × 10^–⁢3^)	0.034
	LDL-C/(HDL-C+DBIL)	2.2 (1.7–2.8)	2.2 (1.7–2.8)	2.6 (1.9–3.4)	<0.001
Clinical diagnosis					
	SCAD	38 (2.8)	32 (2.8)	6 (2.8)	0.986
	UA	1162 (85.0)	997 (86.2)	165 (77.4)	0.136
	NSTEMI	132 (9.6)	99 (8.6)	33 (15.5)	0.125
	STEMI	38 (2.8)	29 (2.5)	9 (4.2)	0.094
Coronary angiography results and treatment					
	Number of L/RIMA	1.0 (1.0–1.0)	1.0 (1.0–1.0)	1.0 (0.0–1.0)	0.017
	Number of SVG	2.0 (1.0–3.0)	2.0 (1.0–3.0)	2.0 (1.0–3.0)	0.778
	Number of other arterial bypass grafts	0.0	0.0	0.0	0.378
	Number of unclosed L/RIMA grafts	1.0 (0.0–1.0)	1.0 (0.0–1.0)	1.0 (0.0–1.0)	0.140
	Number of unclosed SVG grafts	1.0 (0.0–1.0)	1.0 (0.0–1.0)	1.0 (0.0–1.0)	0.966
	Number of native stents	1.0 (1.0–2.0)	1.0 (1.0–2.0)	1.0 (0.5–2.0)	0.797
	Total DES number	1.0 (1.0–2.0)	1.0 (1.0–2.0)	2.0 (1.0–2.0)	0.269
	CABG to PCI time (years)	6.0 (3.0–10.0)	6.0 (3.0–10.0)	7.0 (4.0–10.0)	0.003
Discharge medication					
	Statin	1339 (99.1)	1147 (99.1)	192 (98.6)	0.452
	Aspirin	1343 (99.3)	1149 (99.3)	193 (99.1)	0.580
	P2Y12 receptor antagonist	1341 (99.2)	1147 (99.1)	194 (99.5)	0.553
	ARB	364 (26.9)	306 (26.4)	58 (29.6)	0.344
	ARNI	17 (1.2)	15 (1.3)	2 (0.9)	0.665

MACCE, major adverse cardiac and cerebrovascular events; BP, blood pressure; MI, myocardial infarction; PCI, percutaneous coronary intervention; L/RIMA, left/right internal mammary artery; SVG, saphenous vein graft; CKD, chronic kidney disease; CAD, coronary artery disease; DES, drug-eluting stent; CABG, coronary artery bypass grafting; HDL, high-density lipoprotein; LDL, low-density lipoprotein; TG, triglycerides; TC, total cholesterol; BMI, body mass index; DBIL, direct bilirubin; SCAD, stable coronary artery disease; UA, unstable angina; NSTEMI, non-ST-segment elevation myocardial infarction; STEMI, ST-segment elevation myocardial infarction; ARB, angiotensin II receptor blocker; ARNI, angiotensin receptor-neprilysin inhibitor.

As shown in Table 2, compared with patients in the low ratio group, patients in the medium and high LDL-C/(HDL-C+DBIL) ratio groups were older (*p* = 0.002), had higher weight (*p* < 0.001), and had significantly higher prevalence of diabetes (*p* = 0.030) and MI history (*p* = 0.005). Based on admission examination results, patients in the medium and high LDL-C/(HDL-C+DBIL) ratio groups exhibited significant differences in HDL, LDL, TG, TC, and BMI indices (*p* < 0.001). Finally, in terms of clinical diagnosis type, patients with UA, NSTEMI, and STEMI were more common in the medium and high LDL-C/(HDL-C+DBIL) ratio groups (*p* < 0.001), while patients in the low ratio group tended to have SCAD type (*p* < 0.001).

**Table 2.** **Baseline characteristics and clinical outcomes across different LDL-C/(HDL-C+DBIL) ratios**.

Category		Low ratio group (n = 405)	Medium ratio group (n = 405)	High ratio group (n = 542)	*p*-value (Low vs. Medium)	*p*-value (Medium vs. High)	*p*-value (Low vs. High)	*p*-value
Demographics								
	Age (years)	64 (58–69)	65 (58–70)	66 (61–71)	0.021	0.248	<0.001	0.002
	Male sex, n (%)	118 (29.1)	84 (20.7)	138 (25.5)	0.007	0.103	0.230	<0.001
	Weight (kg)	70 (63–78)	73 (67–80)	75 (66–80)	0.094	0.048	0.037	<0.001
	Systolic BP (mmHg)	129 (117–140)	130 (119–140)	130 (120–140)	0.267	0.340	0.035	0.094
	BMI	25 (23–28)	26 (24–28)	26 (25–29)	0.557	0.455	0.403	<0.001
Risk factors, n (%)								
	Hypertension	287 (70.9)	292 (72.1)	399 (73.6)	0.756	0.606	0.378	0.640
	Hyperlipidemia	404 (99.8)	404 (99.8)	539 (99.4)	0.987	0.640	0.640	0.651
	Diabetes mellitus	175 (43.2)	180 (44.4)	286 (52.8)	0.777	0.022	0.009	0.030
	History of MI	177 (43.7)	205 (50.6)	293 (54.1)	0.057	0.324	0.002	0.005
	Heart failure	23 (5.7)	32 (7.9)	40 (7.4)	0.264	0.805	0.356	0.387
	Stroke history	53 (13.1)	42 (10.4)	78 (14.4)	0.456	0.212	0.757	0.558
	Chronic kidney disease	13 (3.2)	12 (3.0)	27 (5.0)	0.979	0.138	0.195	0.528
	Family history of CAD	34 (8.4)	37 (9.1)	57 (10.5)	0.804	0.511	0.316	0.410
	Number of native stents	1 (1.0–2.0)	1 (1.0–2.0)	1 (1.0–2.0)	0.243	0.672	0.412	0.265
	Total DES count	1 (1.0–2.0)	1 (1.0–2.0)	1 (1.0–2.0)	0.808	0.492	0.686	0.787
	Time from CABG to PCI years	5 (2.0–9.0)	5 (3.0–10.0)	7 (4.0–10.0)	0.006	0.003	0.001	0.002
Admission examination								
	HDL mmol/L	1.1 (1–1.3)	1 (0.9–1.1)	0.9 (0.8–1)	<0.001	<0.001	<0.001	<0.001
	LDL mmol/L	1.6 (1.4–1.9)	2.1 (1.8–2.4)	2.9 (2.4–3.4)	<0.001	<0.001	<0.001	<0.001
	TG mmol/L	1.1 (0.9–1.6)	1.4 (1.1–2)	1.8 (1.3–2.4)	0.357	0.245	<0.001	<0.001
	TC mmol/L	3.2 (2.9–3.7)	3.8 (3.3–4.1)	4.6 (3.9–5.1)	0.025	0.004	<0.001	<0.001
	DBIL mmol/L	3.4 × 10^–⁢3^ (2.6 × 10^–⁢3^–4.7 × 10^–⁢3^)	3.1 × 10^–⁢3^ (2.4 × 10^–⁢3^–4.1 × 10^–⁢3^)	2.6 × 10^–⁢3^ (1.9 × 10^–⁢3^–3.5 × 10^–⁢3^)	<0.001	<0.001	<0.001	<0.001
Coronary angiography results and treatment								
	Number of L/RIMA	1.0 (0.0–1.0)	1.0 (1.0–1.0)	1.0 (1.0–1.0)	0.008	0.372	0.134	0.052
	Number of SVG	2.0 (1.0–3.0)	2.0 (1.0–3.0)	2.0 (1.0–3.0)	0.418	0.335	0.289	0.682
	Number of other arterial bypass grafts	0.0 (0.0–0.0)	0.0 (0.0–0.0)	0.0 (0.0–0.0)	0.174	0.236	0.842	0.055
	Number of unclosed L/RIMA grafts	0.0 (0.0–1.0)	0.0 (0.0–1.0)	0.0 (0.0–1.0)	0.731	0.541	0.212	0.531
	Number of unclosed SVG grafts	1.0 (0.0–2.0)	1.0 (0.0–2.0)	1.0 (0.0–2.0)	0.175	0.379	0.617	0.513
	SCAD	16 (4.0)	7 (1.7)	14 (2.6)	<0.001	<0.001	<0.001	<0.001
	UA	260 (64.2)	347 (85.7)	441 (81.4)	<0.001	<0.006	<0.001	<0.001
	NSTEMI	25 (6.2)	39 (9.6)	66 (12.2)	<0.001	<0.001	<0.001	<0.001
	STEMI	4 (1.0)	12 (3.0)	21 (3.9)	<0.001	<0.001	<0.001	<0.001
Discharge medications								
	Statin	403 (99.5)	402 (99.3)	534 (98.5)	0.932	0.369	0.203	0.113
	Aspirin	403 (99.5)	403 (99.5)	536 (98.9)	0.991	0.514	0.514	0.389
	P2Y12 receptor antagonist	400 (98.8)	403 (99.5)	538 (99.3)	0.451	0.892	0.508	0.465
	ARB	105 (25.9)	109 (26.9)	150 (27.7)	0.811	0.825	0.555	0.551
	ARNI	5 (1.2)	6 (1.5)	6 (1.1)	0.987	0.825	0.912	0.817

BP, blood pressure; MI, myocardial infarction; PCI, percutaneous coronary intervention; CKD, chronic kidney disease; CAD, coronary artery disease; DES, drug-eluting stent; CABG, coronary artery bypass grafting; HDL, high-density lipoprotein; LDL, low-density lipoprotein; TG, triglycerides; TC, total cholesterol; BMI, body mass index; DBIL, direct bilirubin; SCAD, stable coronary artery disease; UA, unstable angina; NSTEMI, non-ST-segment elevation myocardial infarction; STEMI, ST-segment elevation myocardial infarction; ARB, angiotensin II receptor blocker; ARNI, angiotensin receptor-neprilysin inhibitor; L/RIMA, left/right internal mammary artery; SVG, saphenous vein graft.

### 3.2 Long-Term Follow-up Outcomes

According to Table 3, MACCE events occurred in 195 cases (31.1%) of patients who had undergone CABG and subsequently received PCI treatment during the follow-up period. Of these, 40 cases (9.9%) occurred in the low LDL-C/(HDL-C+DBIL) ratio group, 46 cases (11.4%) occurred in the medium ratio group, and 109 cases (20.1%) occurred in the high ratio group. The incidence of MACCE was significantly higher in the medium and high ratio groups (*p* < 0.001) than in the low ratio group. In addition, the three groups’ risks of all-cause mortality, nonfatal stroke, and TVR were comparable, but patients in the high ratio group had a significantly higher risk of cardiac death (*p* = 0.021) and nonfatal myocardial infarction (*p* = 0.003) compared to those in the low and medium ratio groups.

**Table 3.** **Long-term follow-up outcomes across different LDL-C/(HDL-C+DBIL) ratios**.

	Low ratio (n = 405)	Medium ratio (n = 405)	High ratio (n = 542)	*p*-value (Low vs. Medium)	*p*-value (Medium vs. High)	*p*-value (Low vs. High)	*p*-value
MACCE, n (%)	40 (9.9)	46 (11.4)	109 (20.1)	0.724	<0.001	<0.001	<0.001
All-cause mortality, n (%)	26 (6.4)	25 (6.2)	53 (6.8)	0.987	0.065	0.085	0.061
Cardiac mortality, n (%)	25 (6.2)	25 (6.2)	53 (9.8)	0.912	0.065	0.062	0.021
Non-fatal MI, n (%)	13 (3.2)	16 (4.0)	40 (7.4)	0.695	0.041	0.009	0.003
Non-fatal stroke, n (%)	9 (2.2)	13 (3.2)	29 (5.4)	0.509	0.162	0.024	0.110
TVR, n (%)	74 (18.3)	69 (17.0)	124 (22.9)	0.737	0.038	0.103	0.051

MACCE, major adverse cardiovascular and cerebrovascular events; MI, myocardial infarction; TVR, target vessel revascularization; LDL-C, low-density lipoprotein cholesterol; HDL-C, high-density lipoprotein cholesterol; DBIL, direct bilirubin.

**Fig. 3.**
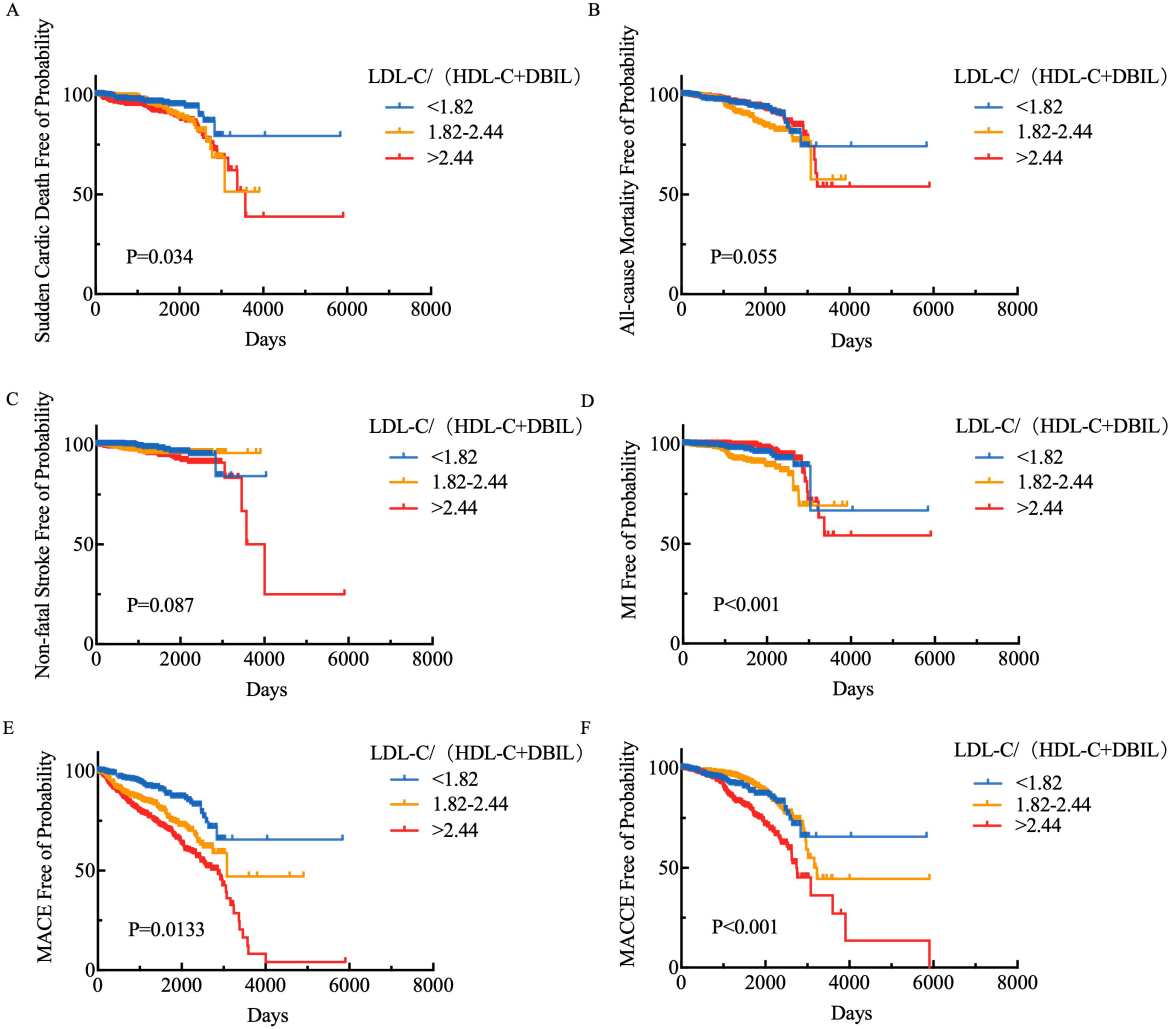
**Kaplan-Meier survival analysis of LDL-C/(HDL-C+DBIL) levels and (A) cardiovascular death, (B) all-cause mortality, (C) non-fatal stroke, (D) myocardial infarction, (E) MACE, and (F) MACCE**. MACCE, major adverse cardiovascular and cerebrovascular events; LDL-C, low-density lipoprotein cholesterol; HDL-C, high-density lipoprotein cholesterol; DBIL, direct bilirubin; MACE, major adverse cardiac events.

Kaplan-Meier survival analysis was performed to compare the cumulative cardiovascular death, all-cause mortality, nonfatal stroke, myocardial infarction, major adverse cardiac events (MACE), and MACCE rates among the low, medium, and high LDL-C/(HDL-C+DBIL) ratio groups. As illustrated in Fig. 3, the cumulative outcomes of cardiovascular death (Log-rank *p* = 0.034), nonfatal myocardial infarction (Log-rank *p* < 0.001), MACCE (Log-rank *p* < 0.001), and MACE (Log-rank *p* = 0.0133) among the three groups demonstrated significant differences, whereas the cumulative outcomes of all-cause mortality (Log-rank *p* = 0.055) and nonfatal stroke (Log-rank *p* = 0.087) did not exhibit significant differences (Fig. 3).

### 3.3 Cox Regression Analysis of Risk Factors for MACCE Occurrence Post-PCI

In the univariate Cox regression analysis, we investigated the association between LDL-C/(HDL-C+DBIL) and MACCE, while incorporating multiple factors such as age, gender, weight, hypertension, hyperlipidemia, diabetes, and BMI. As shown in Table 4, the LDL-C/(HDL-C+DBIL) ratio demonstrated a significant correlation with the incidence of MACCE. The risk of an event was significantly increased for the individual, especially in the high ratio group (hazard ratio (HR) = 1.727, 95% CI: 1.225–2.436, *p* = 0.002). This result suggests that the LDL-C/(HDL-C+DBIL) ratio may serve as an important biomarker with predictive value for MACCE occurrence.

**Table 4.** **Univariable Cox regression analysis for predictors of long-term outcomes**.

		Frequency	HR (95% CI)	*p*-value
Age			1.048 (1.028–1.072)	<0.001
Weight			0.992 (0.979–1.005)	0.247
BMI			0.990 (0.947–1.035)	0.661
Gender				
	Female	328	Reference	
	Male	999	0.850 (0.628–1.151)	0.294
Hypertension				
	No	372	Reference	
	Yes	955	1.053 (0.780–1.442)	0.736
Hyperlipidemia				
	No	5	Reference	
	Yes	1322	0.662 (0.093–4.726)	0.681
Diabetes mellitus				
	No	704	Reference	
	Yes	623	1.353 (1.034–1.772)	0.028
LDL-C/(HDL-C+DBIL)				
	<1.82	405	Reference	
	1.82–2.44	405	1.054 (0.705–1.575)	0.797
	>2.44	542	1.727 (1.225–2.436)	0.002

HR, hazard ratio; CI, confidence interval; BMI, body mass index; LDL-C, low-density lipoprotein cholesterol; HDL-C, high-density lipoprotein cholesterol; DBIL, direct bilirubin.

We applied multivariate Cox regression to adjust for other confounding factors. In Table 5, age, weight, BMI, gender, hypertension, hyperlipidemia, and diabetes were all taken into account in the analysis. LDL-C/(HDL-C+DBIL) remained significantly associated with MACCE after controlling for other variables (HR = 1.33, 95% CI: 1.186–1.193, *p* < 0.001). Following the stratification of LDL-C/(HDL-C+DBIL) into tertiles, individuals in the high ratio group had the highest risk of MACCE, with a hazard ratio of 2.331 (95% CI: 1.585–3.427, *p* < 0.001). While individuals in the medium ratio group showed an increasing trend in MACCE risk, the difference was not significant (HR = 1.188, 95% CI: 0.769–1.835, *p* = 0.439). In conclusion, LDL-C/(HDL-C+DBIL) continues to be an independent predictor of MACCE occurrence even after controlling for other confounding variables.

**Table 5.** **Multivariable Cox regression analysis for predictors of long-term outcomes**.

		Frequency	HR (95% CI)	*p*-value
Age			1.050 (1.026–1.044)	<0.001
Weight			0.983 (0.953–1.014)	0.291
BMI			1.050 (0.955–1.155)	0.315
Gender				
	Female	328	Reference	
	Male	999	1.264 (0.780–2.047)	0.342
Hypertension				
	No	372	Reference	
	Yes	955	0.885 (0.627–1.249)	0.487
Hyperlipidemia				
	No	5	Reference	
	Yes	1322	0.822 (0.087–7.794)	0.865
Diabetes mellitus				
	No	704	Reference	
	Yes	623	1.285 (0.946–1.745)	0.109
LDL-C/(HDL-C+DBIL)				
	<1.82	405	Reference	
	1.82–2.44	405	1.188 (0.769–1.835)	0.439
	>2.44	542	2.331 (1.585–3.427)	<0.001

HR, hazard ratio; CI, confidence interval; BMI, body mass index; LDL-C, low-density lipoprotein cholesterol; HDL-C, high-density lipoprotein cholesterol; DBIL, direct bilirubin.

### 3.4 LDL-C/(HDL-C+DBIL) Levels in Patients Undergoing Native PCI and Graft PCI

**Fig. 4.**
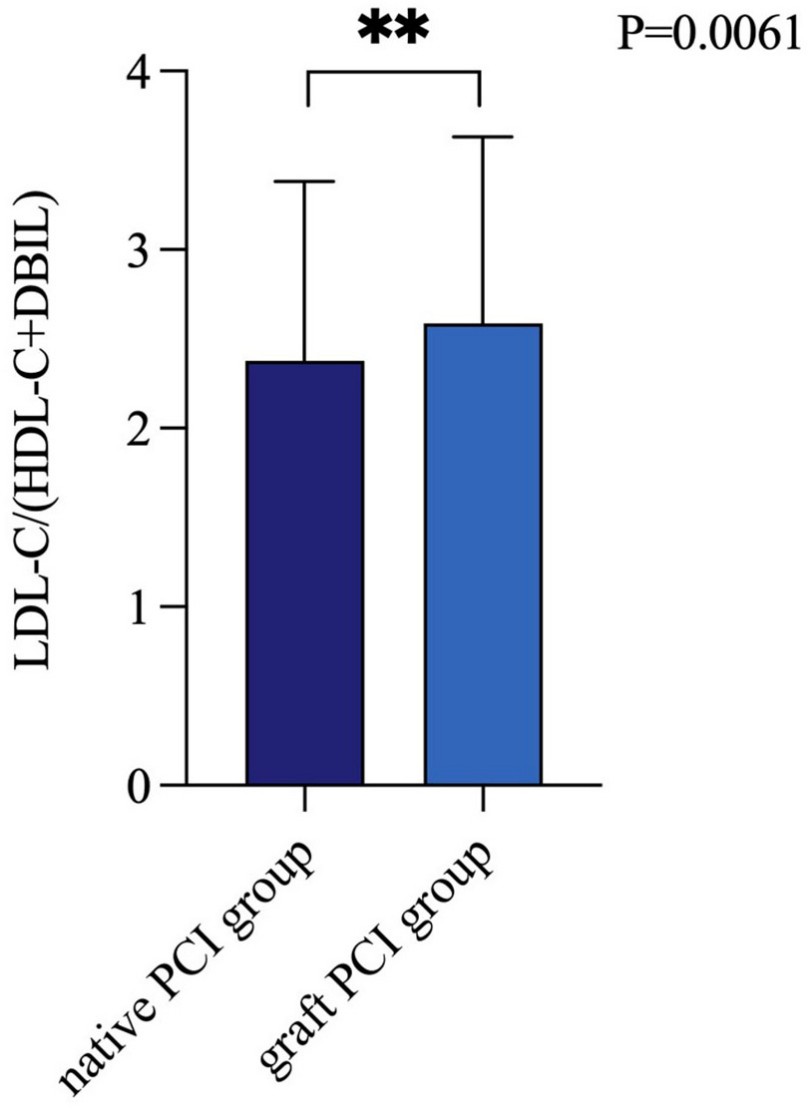
**LDL-C/(HDL-C+DBIL) levels of native vessel PCI and bypass graft PCI**. PCI, percutaneous coronary intervention; LDL-C, low-density lipoprotein cholesterol; HDL-C, high-density lipoprotein cholesterol; DBIL, direct bilirubin. The symbol ** indicates a *p*-value less than 0.01.

To investigate the differential expression of LDL-C/(HDL-C+DBIL) in patients undergoing PCI with grafts versus native vessels, we divided patients into two groups: the native PCI group and the graft PCI group. As depicted in Fig. 4, the LDL-C/(HDL-C+DBIL) ratio was significantly lower in the native PCI group compared to the graft PCI group (*p* = 0.0061).

**Fig. 5.**
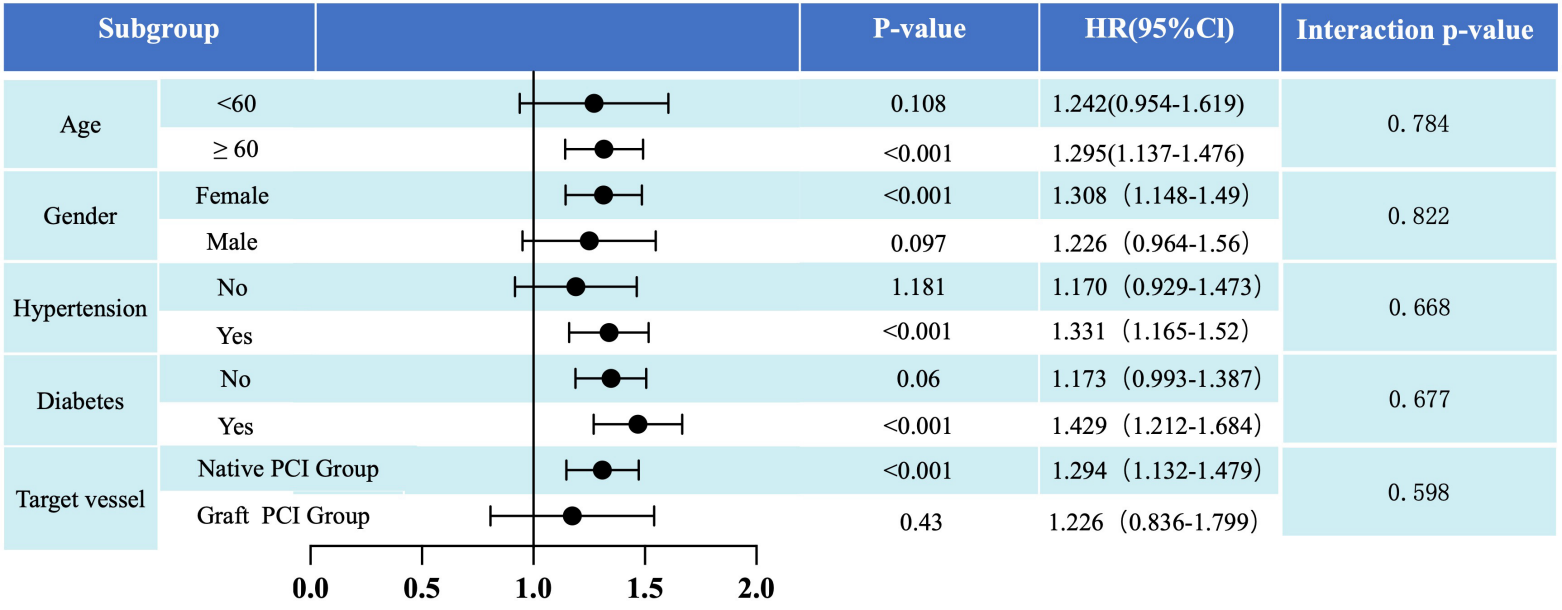
**Post hoc subgroup analysis results for the primary endpoint (risk of MACCE) stratified by age (<60 years vs. ≥60 years), gender (male vs. female), hypertension (absent vs. present), diabetes (absent vs. present), and target vessel selection for stent placement (native vessel vs. bypass graft)**. HR, hazard ratio; CI, confidence interval; PCI, percutaneous coronary intervention; MACCE, major adverse cardiovascular and cerebrovascular events.

Considering variations in baseline risk profiles among patients, this study conducted subgroup analyses to assess the predictive value of LDL-C/(HDL-C+DBIL) for MACCE risk across different baseline levels [age (<60 years vs. ≥60 years), gender (male vs. female), hypertension (present vs. absent), diabetes (present vs. absent), and target vessel for stent placement (*in-situ* graft vs. CABG)]. As illustrated in Fig. 5, there was no significant interaction between LDL-C/(HDL-C+DBIL) and age, gender, hypertension, diabetes, and target vessel for stent placement.

## 4. Discussion

This study employed a large-sample, single-center, observational, retrospective design to investigate the relationship between LDL-C/(HDL-C+DBIL) levels and the incidence of MACCE among patients with prior history of CABG undergoing PCI. LDL-C/(HDL-C+DBIL) level emerged as a significant risk factor, with higher levels correlating with increased incidence of MACCE. Although LDL-C/(HDL-C+DBIL) levels in the native PCI group were significantly lower than those in the graft PCI group, the effect of LDL-C/(HDL-C+DBIL) on MACCE is consistent whether the target vessels are native arteries or grafts.

Serum biochemical factors are considered auxiliary indicators for assessing the presence of atherosclerotic plaques [20]. In 1994, Schwertner *et al*. [21] first reported an inverse relationship between total bilirubin (TBIL) levels and the prevalence of CAD in a cross-sectional study. Subsequent epidemiological studies have consistently demonstrated that low serum bilirubin concentrations are independently associated with an increased risk of CAD, suggesting that bilirubin may have protective cardiovascular effects [22]. The relationship between bilirubin and lipoproteins, particularly in the context of lipid metabolism, remains of great interest in understanding the pathophysiology of atherosclerosis. Bilirubin, a byproduct of hemoglobin degradation, plays a multifaceted role in lipid metabolism. On one hand, bilirubin has been shown to facilitate the dissolution and excretion of TC, thereby reducing LDL levels and increasing HDL content [23]. Elevated levels of small, dense LDL particles and oxidized LDL are known to penetrate the arterial wall, promoting cholesterol crystallization and plaque formation, which are key mechanisms in the development of atherosclerosis and cardiovascular disease [24]. On the other hand, bilirubin serves as a potent endogenous antioxidant, exerting protective effects by reducing the oxidative modification of LDL. The oxidation of LDL is a pivotal process in atherogenesis, as oxidized LDL (oxLDL) triggers endothelial dysfunction, promotes foam cell formation, and induces inflammatory responses in the arterial wall, all of which accelerate the development of atherosclerotic plaques. Bilirubin, through its antioxidant properties, scavenges reactive oxygen species (ROS) and other free radicals that initiate LDL oxidation. By preventing LDL oxidation, bilirubin reduces the formation of oxLDL, thus mitigating its atherogenic potential and the downstream inflammatory cascade [25]. Additionally, HDL, commonly referred to as “good cholesterol”, is recognized for its role in reverse cholesterol transport, antioxidant properties, and anti-inflammatory effects. HDL not only facilitates the removal of excess cholesterol from the arterial wall but also mitigates oxidative stress and inflammation in the vascular endothelium, further protecting against atherosclerotic disease [26]. The combined effects of HDL and bilirubin, particularly when expressed in a composite ratio such as LDL/(HDL+DBIL), may offer a more comprehensive reflection of cardiovascular risk. This ratio could represent the synergistic interplay between HDL’s anti-atherogenic properties and bilirubin’s antioxidant and anti-inflammatory functions, potentially providing a more robust predictor of cardiovascular health.

The majority of patients in this cohort were treated with statins (99.1%), which are known to effectively lower LDL-C and TC levels [27]. Moreover, lipid metabolism and bilirubin metabolism are closely interconnected, as both processes are primarily regulated by the liver. Lipid-lowering therapies, particularly statins, have been shown to elevate bilirubin levels, which in turn may contribute to a reduced incidence of MACCE in patients [28].

In patients with a history of combined CABG, early graft failure, particularly in SVGs, is primarily attributed to surgical factors, including anastomotic stenosis and vascular endothelial injury, which can lead to acute thrombosis, intimal hyperplasia, and fibrosis. Late graft failure is predominantly due to the progression of atherosclerotic lesions, resulting in luminal narrowing or complete occlusion [29, 30]. Furthermore, underlying patient conditions, such as diabetes, hypertension, and hyperlipidemia, accelerate the process of atherosclerosis and create a more unfavorable environment for the grafts. Diabetes, in particular, is associated with an increased risk of graft failure, largely due to its adverse effects on endothelial function and microvascular circulation [31]. Additionally, the choice of graft material—whether arterial or venous—can significantly influence graft patency, with arterial grafts typically exhibiting superior long-term outcomes compared to venous grafts [32]. The duration of surgery can also affect liver function, which plays a key role in processing medications and recovery. Prolonged surgery may lead to hepatic stress, altering drug metabolism and impacting patient recovery [33]. Additionally, medications such as anticoagulants and antiplatelets, used during and after CABG, can affect graft patency and liver function [34]. Chronic medication use may contribute to hepatic dysfunction, influencing cardiovascular disease progression. Therefore, evaluating liver function before and after CABG is crucial when assessing PCI outcomes and making treatment decisions.

The 2018 European Society of Cardiology (ESC) myocardial revascularization guidelines recommend native vessels as the preferred target for intervention in patients with failed grafts [35]. However, there remains controversy regarding the comparative outcomes of PCI using native vessels versus graft vessels. The ARTS-II trial, a randomized controlled study in post-CABG patients, aimed to compare the efficacy of PCI using native vessels versus graft vessels [36]. Over a 5-year follow-up period, there was no significant difference in MACCE between the native vessel PCI group and the graft vessel PCI group, with rates of 36% and 31% respectively. Furthermore, the native vessel PCI group had a slightly higher mortality rate than the graft vessel PCI group, but lower rates of myocardial infarction and repeat revascularization. Similarly, the 2019 EXCEL trial published in the Lancet demonstrated comparable MACCE rates for patients undergoing PCI using native vessels or graft vessels following left main coronary artery stenosis [37]. With 1905 patients enrolled, comprising 948 receiving native vessel PCI and 957 receiving graft vessel PCI, there was no statistically significant difference in MACCE rates between the two groups over a three-year follow-up period (15.4% vs. 14.7% respectively). Additionally, the native vessel PCI group exhibited a slightly higher mortality rate compared to the graft vessel PCI group (5.3% vs. 3.0%), but lower rates of myocardial infarction and stroke. These findings provide valuable insights suggesting that while there may be no significant difference in reducing MACCE between native vessel PCI and graft vessel PCI in post-CABG patients, each approach may offer advantages in specific outcomes. Some studies have begun exploring potential biomarkers such as high-sensitivity C-reactive protein (hs-CRP), cardiac troponin T (cTnT), and B-type natriuretic peptide (BNP) [38, 39, 40, 41]. These biomarkers are closely associated with the development and progression of cardiovascular disease and may guide the choice of intervention. Alternatively, imaging modalities like computed tomography and magnetic resonance imaging can provide specific parameters to assist in treatment decision-making [42]. However, there is currently no definitive biomarker to guide the choice between native vessel PCI and graft vessel PCI in post-CABG patients. The LDL-C/(HDL-C+DBIL) ratio has shown potential in assessing the risk and prognosis of cardiovascular disease, suggesting it as a possible biomarker. This study investigated the differences in LDL-C/(HDL-C+DBIL) ratio between patients undergoing native vessel PCI and graft vessel PCI, and analyzed its relationship with MACCE rates. Results showed a significant difference in LDL-C/(HDL-C+DBIL) ratio between the two groups, with the graft vessel PCI group exhibiting higher ratios, indicating higher cardiovascular risk factors prior to PCI. Further univariate Cox regression analysis and subgroup analysis revealed a positive correlation between LDL-C/(HDL-C+DBIL) ratio and MACCE rates in the native vessel PCI group, suggesting an increase in the ratio was associated with higher MACCE rates. However, in the graft vessel PCI group, an increase in LDL-C/(HDL-C+DBIL) ratio did not show a significant association with cardiovascular MACCE rates. This indicates that the LDL-C/(HDL-C+DBIL) ratio may have different effects on MACCE rates between native vessel treatment and graft vessel treatment. These findings are consistent with previous studies suggesting that MACCE rates may not significantly differ between different treatment groups. We also highlight the potential role of LDL-C/(HDL-C+DBIL) levels in guiding treatment selection and assessing patient prognosis.

## 5. Limitation 

The increasing proportion of CABG procedures observed in recent years raises important questions about the underlying causes of this trend. While some of this increase can be attributed to the growing prevalence of CAD and the aging population, advancements in surgical techniques and patient selection criteria also play a key role. With the improvement in grafting methods, such as the widespread adoption of ITA and the development of hybrid procedures, more patients are eligible for CABG who may have previously been considered inoperable or at high risk for complications [43, 44]. These technical advancements contribute to the expanding role of CABG as a primary intervention for coronary artery disease, thus explaining the increased proportion of cases in clinical settings. The statistical findings presented in this study reflect the expanding indications for CABG and its growing acceptance as a viable treatment option. However, to ensure that these findings are not merely statistical artifacts, it is crucial to evaluate whether the increase in CABG procedures truly translates into improved patient outcomes, particularly in terms of post-operative recovery and long-term survival. This evaluation requires integrating several key factors, including the technical aspects of CABG, surgical duration, the use of specific medications, and the rationale behind the increasing number of CABG procedures. By considering these elements, a more comprehensive understanding of the impact of CABG on patient outcomes can be achieved. The data was collected from past records without randomization. This design may introduce selection bias, which could impact the generalizability of the findings. While the study establishes an association between LDL-C/(HDL-C+DBIL) and MACCE, it does not explore the underlying biological mechanisms or modes of action, making it difficult to draw definitive conclusions about the cause-and-effect relationship between these biomarkers and MACCE. Therefore, the study’s design prevents a deeper insight into how LDL-C/(HDL-C+DBIL) levels influence cardiovascular outcomes, highlighting the need for further research to investigate these mechanisms and validate the observed association. Additionally, an important limitation is the absence of a control group. All participants in this study underwent coronary revascularization (PCI), and there was no comparison with a group of patients who did not undergo the procedure. The lack of a control group makes it difficult to assess whether LDL-C/(HDL-C+DBIL) levels independently contribute to MACCE, or if the observed association is confounded by the effects of coronary revascularization. Including a control group, such as patients who did not undergo revascularization, would allow for a more robust comparison and provide a clearer understanding of the role of these biomarkers, independent of the treatment received. Finally, this study focused on a single-center cohort, which may limit the generalizability of the findings to different populations. To confirm the robustness of the results, future studies should adopt a longer-term, more diverse research design with larger sample sizes and additional control groups, which will help provide a more comprehensive understanding of the role of LDL-C/(HDL-C+DBIL) in cardiovascular disease and MACCE.

## 6. Conclusion

This study found that the levels of LDL-C/(HDL-C+DBIL) were positively correlated with the occurrence of MACCE events in the population, with increasing age and diabetes being closely associated with MACCE event rates. When undergoing PCI again after CABG surgery, the LDL-C/(HDL-C+DBIL) levels had different effects on cardiovascular MACCE event rates depending on the target vessel. In the native vessel PCI group, LDL-C/(HDL-C+DBIL) levels were positively correlated with MACCE event rates, whereas in the graft vessel PCI group, an increase in LDL-C/(HDL-C+DBIL) did not show a significant association with cardiovascular MACCE event rates. LDL-C/(HDL-C+DBIL) levels serve as an important indicator for predicting cardiovascular event risk, particularly in risk assessment prior to PCI treatment, holding significant clinical relevance. These findings offer new directions for the prevention and management of cardiovascular diseases, providing valuable reference for clinical practice and further research endeavors.

## Data Availability

The datasets generated and analyzed during the current study are available from the corresponding author on reasonable request. Additionally, any materials used in the study are available upon request.

## Abbreviations

ACS, acute coronary syndrome; BMI, body mass index; CABG, coronary artery bypass grafting; CAD, coronary artery disease; CRP, C-reactive protein; DBIL, direct bilirubin; HDL, high-density lipoprotein; LDL, low-density lipoprotein; MACCE, major adverse cardiac and cerebrovascular events; MACE, major adverse cardiac events; NSTEMI, non-ST elevation myocardial infarction; PCI, percutaneous coronary intervention; SVGs, saphenous vein grafts; SCAD, stable coronary artery disease; STEMI, ST-elevation myocardial infarction; TC, total cholesterol; TVR, target vessel revascularization; UA, unstable angina.
