# Erratum: Yan et al. Association Between Serum Lipid-Bilirubin Ratio and Clinical Prognosis Among Patients Undergoing Percutaneous Coronary Intervention After Coronary Artery Bypass Grafting. Reviews in Cardiovascular Medicine. 2025; 26(11): 39827

**DOI:** 10.31083/RCM57380

**Published:** 2026-08-24

**Authors:** Xu Yan, Muhib Ur Rehman, Qiuxuan Li, Zhiqiang Yang, Lixia Yang, Zhijian Wang, Yujie Zhou

**Affiliations:** ^1^Department of Cardiology, Beijing Anzhen Hospital Affiliated to Capital Medical University, 100029 Beijing, China

The authors wish to make the following correction to this paper [1]:

During the production process, the name of the second author was incorrectly published as “Muhib ur Reheman”. The correct name is “Muhib Ur Rehman”. The author list has been corrected accordingly.

This has been corrected as of August 3, 2026. The publisher apologizes for this error.
